# Pre-dialysis patients' perceived autonomy, self-esteem and labor participation: associations with illness perceptions and treatment perceptions. A cross-sectional study

**DOI:** 10.1186/1471-2369-11-35

**Published:** 2010-12-08

**Authors:** Daphne L Jansen, Diana C Grootendorst, Mieke Rijken, Monique Heijmans, Ad A Kaptein, Elisabeth W Boeschoten, Friedo W Dekker

**Affiliations:** 1 NIVEL, Netherlands Institute for Health Services Research, P.O. Box 1568 3500 BN Utrecht The Netherlands; 2 Department of Clinical Epidemiology, Leiden University Medical Center, P.O. Box 9600 2300 RC Leiden The Netherlands; 3 Unit of Psychology, Leiden University Medical Center, P.O. Box 9600 2300 RC Leiden The Netherlands; 4 Hans Mak Institute, Koningin Wilhelminalaan 29-B 1411 EL Naarden The Netherlands

## Abstract

**Background:**

Compared to healthy people, patients with chronic kidney disease (CKD) participate less in paid jobs and social activities. The aim of the study was to examine a) the perceived autonomy, self-esteem and labor participation of patients in the pre-dialysis phase, b) pre-dialysis patients' illness perceptions and treatment perceptions, and c) the association of these perceptions with autonomy, self-esteem and labor participation.

**Methods:**

Patients (N = 109) completed questionnaires at home. Data were analysed using bivariate and multivariate analyses.

**Results:**

The results showed that the average autonomy levels were not very high, but the average level of self-esteem was rather high, and that drop out of the labor market already occurs during the pre-dialysis phase. Positive illness and treatment beliefs were associated with higher autonomy and self-esteem levels, but not with employment. Multiple regression analyses revealed that illness and treatment perceptions explained a substantial amount of variance in autonomy (17%) and self-esteem (26%). The perception of less treatment disruption was an important predictor.

**Conclusions:**

Patient education on possibilities to combine CKD and its treatment with activities, including paid work, might stimulate positive (realistic) beliefs and prevent or challenge negative beliefs. Interventions focusing on these aspects may assist patients to adjust to CKD, and ultimately prevent unnecessary drop out of the labor market.

## Background

Chronic renal failure, also referred to as end-stage renal disease (ESRD; chronic kidney disease (CKD) stage V), is a permanent condition which requires renal replacement therapy (peritoneal dialysis, haemodialysis or transplantation) to maintain life. At the end of 2005, approximately 1,9 million people were receiving renal replacement therapy worldwide [1]. In January 2006, 12,038 people in the Netherlands received renal replacement therapy (737 people per million Dutch residents) [2]. ESRD is associated with specific disease and treatment aspects. Patients with ESRD often experience physical symptoms such as fatigue, pain, cramps and itching [3]. Furthermore, patients are extremely dependent on treatment and the treatment itself - dialysis in particular - places substantial behavioral and psychosocial demands on the patient. Neto et al. [4] showed that the quality of life of ESRD patients is already lowered at the initiation of dialysis treatment, which was clearly evidenced in the role limitations due to physical function and emotional function aspects. Various studies demonstrated lowered quality of life of patients with ESRD in a later phase of the dialysis treatment compared to general population samples [5-9].

Results of a literature study revealed that people with chronic renal insufficiency experience difficulties in participating in various domains of life, such as paid work, sports and other social and leisure activities [10]. It seems in particular difficult to combine dialysis treatment with a paid job: several studies found labour participation rates around 24% in dialysis patients aged below 65 [11-13]. It is notable that people with CKD who are being prepared for renal replacement therapy (pre-dialysis patients; CKD stage IV) already experience work-related problems. Results from a Dutch study showed that patients mainly drop out the labor market before the start with dialysis treatment: at the start of the treatment only 35% of the patients, aged 18 to 64 years, had a paid job compared to 61% in the general population in 1997, the year the study was carried out [14]. A Swedish study among pre-dialysis patients and patients on dialysis demonstrated that around 30% of the pre-dialysis patients and more than 50% of the dialysis patients reported stressors with respect to work and leisure time [15].

Restrictions with regard to labor participation can have serious drawbacks for a person's well-being. Work is generally good for physical and mental health and well-being, and unemployment is associated with negative health effects [16]. Moreover, participation in general is important for feelings of autonomy and self-esteem. According to Self-Determination Theory (SDT) autonomy is one of the basic psychological needs for optimal functioning [17]. Reis et al. [18] found that variations in the fulfilment of autonomy independently predicted variability in daily well-being. Factors in the person or situation that facilitate autonomy are thus expected to enhance well-being, whereas factors that detract from fulfilment of this need will undermine well-being. In the SDT view, self-esteem is a derivative or by-product of need dynamics. When the fulfilment of the need for autonomy is hindered, one's experience of self-worth is also damaged, leading to either insecure or low self-esteem [19]. The feelings of self-worth depending on a person's experience, is referred to as state self-esteem. Research showed that high as well as stable self-esteem are associated with greater psychological well-being [20].

In light of these findings it is important to uncover the factors that influence feelings of autonomy, self-esteem and labor participation in patients with CKD. Socio-demographic factors (e.g. age, educational level) and medical factors (e.g. severity of the health condition, type of treatment) obviously determine the extent to which patients with CKD participate in paid jobs. Besides these factors, psychological factors may be important for labor participation and perceived autonomy as well, in particular the way patients view their illness and treatment [10]. Braun Curtin et al. [11] demonstrated that employed dialysis patients did not feel limited by their health in the hours they worked or the kind of work in which they could engage. Unemployed patients on the other hand, perceived their illness as a barrier to work. These findings are important since both patient groups did not differ with respect to objective health indicators.

Patients' beliefs about their illness are the central concepts of the Common Sense Model (CSM), which is a self-regulation model of health threat [21,22]. This model aims to explain patients' responses to illness from the cognitive representations patients hold about their medical condition. Five domains of illness representations have been identified: (1) the identity or label (e.g. 'renal disease') with associated emotions ('it makes me afraid') and symptoms ('tiredness', 'itching'); (2) timeline, reflecting patients' expectations about the duration of the condition and its characteristic course (acute, chronic, or episodic), (3) cause, reflecting patients' ideas about how one gets the disease (e.g. by stress or bad luck), (4) beliefs about the cure or controllability of the disease, and (5) patient's expectations about the physical, social, economic and emotional consequences of the disease. The CSM predicts that these cognitions are directly related to coping and via coping to adaptive outcomes such as quality of life. Furthermore, as treatment constitutes a major part of the experience of any chronic illness, it should be anticipated that patients also develop their beliefs regarding treatment or engage in treatment appraisals and evaluations that complement illness perceptions [23-25]. Recent studies found relationships between dialysis patients' illness representations and well-being [9], mortality [26], and dialysis and renal transplant patients' representations about their illness and treatment and health related quality of life [27]. Our research team recently conducted a study among patients on dialysis [12] and the results showed that patients' illness and treatment perceptions significantly contributed to the explained variance in both perceived autonomy and state self-esteem, after controlling for socio-demographic and clinical characteristics. Beliefs about greater personal control over the disease, less perceived impact of the illness and treatment on daily life, and less concern about the illness were important determinants. Contrary to our expectations, no significant associations were found between illness perceptions, treatment perceptions and labor participation. This may be caused by the fact that the working age group (18-64 years) of dialysis patients was small (N = 62). An additional explanation might be that patients who are on dialysis do not value a paid job as that important anymore, i.e. performing paid work does not contribute to their feelings of autonomy and self-esteem. Patients in this stage of the illness are aware of the fact that they are seriously ill, and therefore other life domains might have become more important. The aim of the present study was to investigate the associations of the illness perceptions and treatment perceptions with perceived autonomy, self-esteem and labor participation among pre-dialysis patients. It is expected that the associations with employment are stronger in this group of patients compared to patients on dialysis, since paid work is presumably more important and relevant to patients in an earlier phase of the illness. The following research questions were formulated:

1) To what extent do pre-dialysis patients experience autonomy, and state self-esteem, and to what degree do these patients participate in the work domain?

2) Which perceptions do pre-dialysis patients have about their illness and treatment?

3) To what extent are illness perceptions and treatment perceptions of pre-dialysis patients related to perceived autonomy, state self-esteem and labor participation?

## Methods

### Participants and procedure

Pre-dialysis patients who were participating in the PREPARE-2 study, were invited to participate in the present cross-sectional study. PREPARE-2 is a prospective observational study started in 2004. At the end of 2006, PREPARE-2 was operating in 18 pre-dialysis outpatient clinics in community and university hospitals throughout the Netherlands. Patients with stage IV CKD (severe CKD) aged 18 years or older who were treated by a nephrologist and recently (within the previous six months) referred to pre-dialysis care were eligible for inclusion. All patients had to be suitable for renal replacement therapy. Patients with chronic transplant dysfunction were excluded from the study if the transplant was within the previous year. Clinical (medical records) and quality of life (self report) data are collected at inclusion and every six months thereafter until start of dialysis, transplantation, end of study or death, whichever occurs earliest. All patients gave written informed consent. The PREPARE-2 study was approved by the institutional review boards of all participating hospitals.

For the present study, data were collected in 2006 by means of an additional survey sent in two phases to all patients recruited at that time: in the period July-September 2006 to 123 patients and in November-December 2006 to another 62 newly recruited patients. Patients completed a paper questionnaire at home. Of the 185 patients who received the questionnaire, 109 returned the questionnaire (response rate 59%).

### Measures

#### Perceived autonomy

Perceived autonomy was assessed with three items derived from the autonomy scale of the CASP-19 [28]. One item 'My health stops me from doing the things I want to do' (reverse scored) was used as an indicator for 'health related autonomy'. The other two items were combined on the basis of their high factor loadings on one factor (both factor loadings: 0.86, variance explained: 74%) to assess 'global autonomy' ('I can do the things that I want to do', 'I feel that I can please myself what I can do'). Items were scored on a 4-point scale (0 = never, 1 = sometimes, 2 = not so often, 3 = often). Global autonomy scores are expressed as average scores based on the two items. Higher scores on both measures signify a higher level of perceived autonomy.

#### Sate self-esteem

State self-esteem was measured with the Current Thoughts Scale [29], which comprises 20 items (e.g. 'I am worried about what other people think of me' (reverse scored)). Items were rated on a 5-point scale (1 = not at all, 2 = a little bit, 3 = somewhat, 4 = very much, 5 = extremely). Scores are summed across individual ratings with higher scores representing a higher level of state self-esteem. The Cronbach's alpha for the scale in the current study was 0.86. The scale has proven to be psychometrically sound and has a high degree of construct validity [29].

#### Labor participation

Labor participation was defined in conformity with Statistics Netherlands (CBS), as performance of paid work for at least 12 hours per week. A full-time employment in the Netherlands consists usually of 36 working hours. It should be noted that employers in the Netherlands must pay at least 70% of the salaries of sick employees for the first two years, consequently people who are on long-term sick leave are in fact still employed.

In addition people were asked to indicate whether performing paid work was of personal importance on a 7-point scale (1 = not important at all to 7 = extremely important).

#### Illness perceptions

Illness perceptions were assessed using the Brief Illness Perception Questionnaire [30], which is a brief version of the Revised IPQ [31]. The questionnaire includes eight items scored on an 11-point scale, ranging from 0 to 10. Each item assesses a cognitive and emotional illness representation dimension. A higher score on the eight dimensions implies greater perceived influence of the illness upon life ('consequences'), a stronger belief in a chronic time course ('timeline'), greater perceived personal control over the illness ('personal control'), greater perceived treatment control over the illness ('treatment control'), greater experience of severe symptoms as a result of the illness ('identity'), greater feelings of concern about the illness ('concern'), better understanding of the illness ('understanding') and a stronger emotional response to the illness ('emotional response'). A ninth open-ended response item assessing the patients' causal representation was not included in the study. The Brief IPQ has proven to be a reliable and valid measure of illness perceptions in a variety of illness populations [30].

#### Treatment perceptions

Treatment perceptions were assessed with the Treatment Effects Questionnaire (TEQ; originally developed as the IEQ-Tx by Greenberg and Peterson [32]; adapted by Griva et al. [27]). The TEQ consists of 20 items (e.g. 'My life revolves around this treatment'), scored on an 8-point scale (0 = strongly disagree to 7 = strongly agree). Scores are summed across individual ratings with higher scores indicating greater perceived disruption from the treatment. The TEQ has been used in a study with ESRD patients [27]. The Cronbach's alpha for the scale in the current study was 0.94.

#### Background variables

Background characteristics included age, gender, living status (living with versus without a partner), educational level (highest level of completed education, classified as low (primary education, lower secondary and lower vocational education), moderate (intermediate secondary and intermediate vocational education) and high (higher vocational education and university)) and number of comorbid diseases (based on the presence of diabetes mellitus type 2, hypertension, cerebrovascular accident, vascular problems, ischemic heart disease, and heart failure).

### Statistical analysis

Descriptive statistics were computed to describe the extent to which pre-dialysis patients experience autonomy and state self-esteem, participate in the work domain, and rate work as personally important. Relationships of the background characteristics with autonomy, state self-esteem and labor participation were assessed by use of analysis of variance (ANOVA) and Chi-square tests.

Descriptive statistics were computed to describe patients' illness and treatment perceptions. Relationships of the background characteristics with illness and treatment perceptions were assessed by means of ANOVA. Associations between illness perceptions and treatment perceptions on the one hand and autonomy, state self-esteem and labor participation on the other hand were analysed by means of Pearson's correlation coefficients and Student's t-test. Furthermore, multiple linear regression analyses were performed, using the enter method, to examine the relationship between illness and treatment perceptions on the one hand and perceived autonomy and state self-esteem on the other hand, controlling for background characteristics. Two blocks of variables were entered separately; block 1: Background variables (age, gender, educational level, number of comorbid diseases); block 2: Illness and treatment perceptions variables. To perform regression analyses, the missing values on the comorbidity variable were replaced by the mean number of comorbid diseases computed over the total study group.

## Results

### Patients

Characteristics of the total study group are outlined in Table 1. Approximately two-thirds of the patients were male. Patients had a mean age of 64 years (SD = 14.9 years). The patients' number of comorbid conditions ranged from 0 to 5, with 46% of the patients suffering from two or more comorbid conditions. Differences between the responders and the non-responders with respect to age, gender, and number of comorbid diseases were examined and no significant differences were found.

**Table 1:** Background characteristics of participating patients

	Total group	
Gender - N (%)		
Male	69	(64)
Female	39	(36)
Unknown	1	
Age, mean in years (SD)	64.3 (14.9), range: 19-92	
Age, in groups - N (%)		
18 - 49 years	21	(20)
50 - 64 years	24	(22)
≥ 65 years	63	(58)
Unknown	1	
Educational level - N (%)		
Low	46	(43)
Moderate	45	(43)
High	15	(14)
Unknown	3	
Living status - N (%)		
Living with a partner	69	(64)
Living without a partner	38	(36)
Unknown	2	
Number of comorbid diseases, mean (SD)	1.5 (1.2), range: 0-5	
Number of comorbid diseases, in groups - N (%)		
No comorbid diseases	23	(24)
One comorbid disease	28	(30)
Two or more comorbid diseases	44	(46)
Unknown	14	

### Perceived autonomy

The mean global autonomy score of the total sample was 1.9 (SD = 0.8, range scores = 0-3, N = 100) and the mean score on the health related autonomy item was 1.4 (SD = 1.0, range scores = 0-3, N = 101). No significant differences in autonomy scores were found according to age, gender, educational level, living status and number of comorbid diseases.

### State self-esteem

The mean state self-esteem score of the total patient group was 78.2 (SD = 10.3, range scores = 46-98, N = 104). ANOVA analysis showed that high educated patients had higher state self-esteem compared to patients with a low and moderate educational level (F (2, 100) = 3.50, p = 0.03). No associations were found between state self-esteem and the other background characteristics.

### Labor participation

Of the total group of patients, forty-five people were of working age (18-64 years), with a mean age of 50 years (10.7 years). Twenty-three patients (51%) performed paid work for at least 12 h per week. Patients who worked (at least 12 h per week) were working for 34.7 h per week on average (range: 20-60 h per week). The majority worked in the 'industry, mineral extraction, construction' sector (N = 4), 'services provision' sector (N = 4), 'health and welfare' sector (N = 4), and the 'commercial' sector (N = 3). Eighteen people aged 18 to 64 years (40%) were not employed (for at least 12 h per week) and the employment status of four people (9%) was unknown. Of those who were not employed, 15 people indicated that they were employed in the past (for at least 12 h per week). Most of them had worked in the 'industry, mineral extraction, construction', 'commercial' and 'health and welfare' sector (N = 12). The results of the ANOVA analysis showed that among the patients of working age, employed patients were significantly younger than unemployed patients (F (1, 39) = 4.19, p = 0.047). No significant differences were found with regard to the other background variables.

Working age patients' mean importance rating score with respect to performing paid work was 5.1 (2.4), which indicates that patients regard paid work as considerably important. Unemployed patients rated the importance of performing paid work with a mean score of 3.4 (2.3) and employed patients' mean importance score was 6.7 (0.6).

### Illness and treatment perceptions

Mean illness perceptions scores indicate that pre-dialysis patients believe that their illness is chronic (timeline). Furthermore, patients experience a moderate amount of physical symptoms from their illness (identity) and believe their illness affects their daily life to a rather large extent (consequences). On the other hand, patients experience rather little disruption of daily life from their current treatment (in most cases medication and diet restrictions). Patients are fairly concerned about their illness, however, do not believe strongly that their illness affects them emotionally. In addition, patients believe that they understand their illness rather well, and consider their illness to be positively influenced by the treatment they receive (treatment control), yet believe that they themselves have rather little control over their illness (personal control) (Table 2). ANOVA analysis showed that patients in the different age groups differed with respect to their beliefs about the timeline of the illness (Welch F (2, 29.35) = 4.36, p = 0.02). Games-Howell post hoc-tests, however, did not point to significant differences between two or more groups in particular. High educated patients believed that their emotional state was less affected by their illness compared to low and moderate educated patients (F (2, 100) = 3.31, p = 0.04). No differences were found with respect to gender, living status and number of comorbid diseases.

**Table 2:** Mean scores and standard deviations of illness and treatment perceptions of pre-dialysis patients (total group)

	N	Range scale	Range scores	M (SD)
*Illness and treatment perceptions*				
Consequences	105	0-10	0-10	6.7 (2.5)
Timeline	104	0-10	0-10	9.3 (1.7)
Personal control	103	0-10	0-10	4.7 (2.9)
Treatment control	103	0-10	0-10	6.8 (2.9)
Identity	103	0-10	0-10	5.2 (2.9)
Concern	104	0-10	0-10	6.9 (2.7)
Understanding	102	0-10	0-10	7.3 (3.1)
Emotional response	104	0-10	0-10	5.0 (3.1)
Treatment disruption	94	0-140	0-125	38.8 (25.9)

The Pearson's correlation coefficients between the illness perceptions and treatment perceptions are depicted in Table 3. As patients experience a large impact from the illness on daily life, they believe that their treatment disrupts their life, experience more physical complaints from the illness, believe they have little personal control over the illness, are worried about their illness and feel that their illness affects them emotionally. As patients experience disruption from the treatment, they experience more consequences and symptoms from the illness, feel that their illness cannot be controlled by medical treatment, are concerned about their illness and experience a large emotional impact due to the illness. Personal and treatment control beliefs are positively interrelated and both associated with less concern.

**Table 3:** Pearson's correlations between illness perceptions, treatment perceptions and perceived autonomy, state self-esteem (total group)

	1	2	3	4	5	6	7	8	9
1. Consequences									
2. Timeline	.11								
3. Personal control	-.33**	-.05							
4. Treatment control	-.17	.06	.44***						
5. Identity	.65***	.07	-.18	-.16					
6. Concern	.59***	.09	-.30**	-.20*	.49***				
7. Understanding	.13	-.01	.18	.21*	.23*	.03			
8. Emotional response	.58***	.04	-.20*	-.14	.44***	.62***	.24*		
9. Treatment disruption	.45***	.01	-.10	-.23*	.47***	.45***	.12	.56***	

Global autonomy	-.36***	.00	.22*	.29**	-.37***	-.30**	-.01	-.37***	-.42***
Health related autonomy	-.44***	-.12	.16	.15	-.34***	-.28**	-.02	-.24*	-.21
State self-esteem	-.39***	.04	.20*	.21*	-.37***	-.44***	.03	-.49***	-.48***

* p < .05; * * p < .01; *** p < .001

### Associations between independent and dependent variables

Pearson's correlation coefficients between illness perceptions and treatment perceptions and perceived autonomy and state self-esteem showed that stronger positive beliefs about the illness and treatment are related to higher levels of perceived autonomy and state self-esteem (Table 3). Within the working age group, the associations between the illness and treatment representations and labor participation were investigated by means of Student's t-test and the results demonstrated no significant associations (Table 4).

**Table 4:** Differences in mean illness and treatment perceptions scores between employed and unemployed patients of working age (18-64 years)

	Employed		Unemployed				
				
	N	M (SD)	N	M (SD)	t	Df	P
Consequences	22	6.7 (2.3)	18	6.8 (2.9)	.129	38	0.9
Timeline	22	8.5 (2.6)	18	8.6 (2.3)	.071	38	0.9
Personal control	21	4.9 (2.8)	18	3.7 (3.2)	-1.229	37	0.23
Treatment control	21	6.8 (2.0)	18	5.6 (3.5)	-1.331	26.072	0.20
Identity	22	5.1 (2.9)	18	5.4 (3.2)	.365	38	0.72
Concern	22	7.0 (2.8)	18	7.0 (2.7)	.051	38	0.9
Understanding	21	7.1 (3.0)	18	7.1 (3.2)	-.032	37	0.9
Emotional response	22	5.6 (2.7)	18	4.5 (3.2)	-1.123	38	0.27
Treatment disruption	21	34.7 (22.9)	17	44.3 (31.3)	1.095	36	0.28

Regression analysis was conducted with global autonomy being the dependent variable. The results showed that the background variables accounted for only 3% of the variance in global autonomy (Table 5). In this model, fewer comorbid diseases were significantly associated with higher levels of global autonomy. Adding the illness and treatment perceptions to the model (block 2) the percentage of explained variance significantly increased to 20%. None of the included variables reached the level of significance, though less perceived disruption from treatment was close to significance (p = 0.054). The results of the regression analysis with health related perceived autonomy being the dependent variable, demonstrated that the background variables and illness and treatment perceptions variables did not explain any substantial amount of variance (adjusted R^2 ^= 3,5%; data not shown).

**Table 5:** Multiple linear regression models for the association between the independent variables and perceived global autonomy in pre-dialysis patients (total group)

	Model 1 (block 1) (N = 88) Beta	Model 2 (block 1+2) (N = 88) Beta
Block 1: Background characteristics		
Age in years	.14	.02
Gender (ref: male)	.01	- .04
Educational level (ref: low)		
Moderate	- .06	- .07
High	.03	- .07
Number of comorbid diseases	- .27*	- .17

Block 2: Perceptions		
Consequences		- .06
Personal control		.08
Treatment control		.12
Identity		- .22
Concern		.19
Emotional response		- .14
Treatment disruption		- .25

Adjusted R^2^	0.03	0.20**
F change model	1.51	3.52**

* p < .05; * * p < .01; *** p < .001

Finally, we performed regression analysis with state self-esteem being the dependent variable. The results showed that the background variables explained 5% of the variance. In the second model, in which the illness and treatment perceptions were added, the percentage of explained variance increased by 26%, to 31%, with less perceived disruption from the treatment being the only significant predictor of state self-esteem (Table 6).

**Table 6:** Multiple linear regression models for the association between the independent variables and state self-esteem in pre-dialysis patients (total group)

	Model 1 (block 1) (N = 91) Beta	Model 2 (block 1+2) (N = 91) Beta
Block 1: Background characteristics		
Age in years	.18	.07
Gender (ref: male)	- .06	- .08
Educational level (ref: low)		
Moderate	.00	- .04
High	.18	.09
Number of comorbid diseases	- .16	- .05

Block 2: Perceptions		
Consequences		.07
Personal control		.18
Treatment control		- .06
Identity		- .18
Concern		- .11
Emotional response		- .13
Treatment disruption		- .31*

Adjusted R^2^	0.05	0.31***
F change model	1.88	5.57***

* p < .05; * * p < .01; *** p < .001

## Discussion

The first aim of the study was to investigate the extent to which pre-dialysis patients experience feelings of autonomy and self-esteem, and participate in the work domain. Secondly, we wished to explore the content of patients' illness and treatment perceptions, and whether these perceptions are related to patients' perceived autonomy, state self-esteem and labor participation.

The mean age of the study group (64 years) and the gender distribution (64% male) corresponds with pre-dialysis patients and patients starting dialysis in the Netherlands [33,34]. By comparing the mean scores on the autonomy measures of the total group with the answer scale, the results indicate that patients feel less autonomous because of their health condition or otherwise. In spite of this, most patients reported a high level of self-esteem. The autonomy and self-esteem levels of the pre-dialysis patients are slightly higher than the reported levels by patients on dialysis [12].

Looking at the mean illness and treatment perceptions of pre-dialysis patients it is noticed that patients are quite worried about their illness (M = 6.9) and believe that they themselves have rather little control over their illness (M = 4.7). To compare, patients on dialysis reported mean levels of 6.3 on the 'concern' dimension and 4.9 on the 'personal control' dimension [12]. In a study of Broadbent et al. people with diabetes and people with asthma reported higher mean levels of personal control (M = 6.7) [30]. Feelings of personal control are important for dialysis patients' quality of life [35,9]. Personal control over the illness refers to the feeling that one can influence the course of the illness and one can fit the disease and treatment into daily life. In order to manage their illness pre-dialysis patients obviously are dependent on treatment. However, this does not indicate that there are no possibilities for personal control. It is of great importance that pre-dialysis patients practice self care behaviors, such as following diets and performing daily exercise in order to optimise their health condition [36]. However, patients in this stage of the illness got the news that they have to start with renal replacement therapy in the near future, which indicates that despite of their self care activities they apparently were not able to remain sufficient renal function. This knowledge might have a negative effect on patients' personal control beliefs.

On the whole, the correlation analyses demonstrated that as patients hold more positive beliefs about their illness and their current treatment, they perceive more autonomy (both global and health related) and have a higher self-esteem. In light of these findings it is important to point out the difference between the construct of personal control and the construct of autonomy, since autonomy is often incorrectly equated with ideas of internal locus of control [37,38]. Beliefs of personal control reflect individuals' beliefs regarding the extent to which one feels that one can control or influence an outcome, for example one's illness. However, people are autonomous when they act in accord with their authentic interests or integrated values and desires [17,37-39]. To make the distinction more explicit, a person can experience control over carrying out a walking program, but not feel intrinsically motivated, and thus do not act in accordance with his/her own values.

The regression analyses revealed that the illness and treatment perceptions explain a substantial amount of variance in predicting both global autonomy and state self-esteem after controlling for background characteristics. These results illustrate that less perceived disruption by the treatment upon life is a significant predictor of state self-esteem. The findings furthermore suggest that less perceived impact of the treatment upon life is an important determinant of global autonomy as well. Treatment in the pre-dialysis phase in most cases includes taking pharmacotherapy and following a diet. Although these treatments are far less disruptive than dialysis treatment, the findings show that treatment already is a significant theme in this stage of the illness. Illness representations are considered to be constantly updated as new experiences and knowledge are acquired [22]. In this transition phase of treatments, in which patients receive information on all available renal replacement therapies, it therefore can be expected that patients are more occupied with treatment in general, both their current treatment as well as their future treatment.

It should be noted that a large amount of variance remained unexplained. This indicates that other factors are of influence as well, for example the extent to which people in the patient's close environment, like the patient's partner or care providers, support the patient. Moreover, health related perceived autonomy could not be predicted by the illness perceptions and treatment perceptions. An explanation for this finding might be that patients are inclined to interpret 'health' as 'physical health'. At this stage of the illness, the renal disease - in most cases - will however not be associated with severe physical symptoms, which is also reflected by the mean score on the 'identity' dimension.

Because of the relatively old age of the study group (M = 64 years), only 45 patients (42%) were of working age (18-64 years). Fifty-one percent of the patients aged between 18 and 64 years performed paid work for at least 12 h per week, which is a higher percentage compared to dialysis patients; 24% [12], though considerably lower than that of the general Dutch population between the ages of 15-64 years; 65% [40]. Thus, as suggested by Van Manen et al. [14], drop out of the labor market already occurs before patients start with dialysis treatment. Furthermore, the results show that, despite of their health condition, patients of working age place relatively high importance on carrying out a paid job. These findings point to importance and necessity of work related assistance in an early stage of the illness process. We wish to mention here that the average age of the working age group (18-64 years) was rather high (50 years) and 53% of the working age group was 50 years or older. To put this into perspective, in 2006, 32% of the Dutch people aged 20-64 years were 50-64 years [40]. Notwithstanding that, our results suggest that labour participation in pre-dialysis patients is indeed lower than in the general population. Because of the small number of patients aged 18-64 years, we could not investigate the relationships between patients' perceptions of their illness and current treatment on the one hand and employment on the other hand more thoroughly. The findings, however, do show some trends: employed patients perceive their treatment as less disruptive and their illness as better controllable by self care and medical care than unemployed patients.

A limitation of this study is the replacement of the missing values on the variable comorbidity with the mean value of the total study group. Mean substitution preserves the mean of a variable's distribution; however, mean substitution typically distorts other characteristics of a variable's distribution (i.e., variance, median) [41]. In spite of this we decided to substitute the missing values by the mean in order to be able to make maximal use of the data of all our cases. Another issue to note is the use of single-item measures in order to minimize the burden on respondents. Single-item measures are sometimes seen as less psychometrically sound than multiple-items. However, several studies show that single-item measures and their multiple counterparts are comparable [42,43]. Moreover, Gardner et al. [42] demonstrate that a well-developed single item measure can be appropriate in avoiding common methods variance, which is often a problem with psychological measures that require respondent self-reports of attitudes, beliefs, perceptions, and the like. Furthermore, it is noteworthy that the present study had a cross-sectional design which means that no conclusions can be drawn regarding the causality of the observed relationships. Notwithstanding this limitation, our results suggest that the beliefs pre-dialysis patients hold about their illness and treatment are important factors for patients' sense of (global) autonomy and self-esteem. Finally, it should be noted that the study sample was rather small (N = 109), as well as the working age sample (N = 45). Consequently there was little statistical power to demonstrate relationships between perceptions and labor participation in particular. Future research should take this issue into account. It would be worthwhile to investigate these relationships once again in a larger sample of pre-dialysis patients.

## Conclusions

In light of the findings it seems important that patients with severe CKD are educated by a multi-professional team, comprising of nephrologists, dialysis nurses as well as employment experts and social workers, on the possibilities to combine CKD and its treatment with daily activities, including work. By means of education, positive (realistic) beliefs might be stimulated and negative beliefs may be prevented or challenged. This might contribute to a greater sense of autonomy and self-esteem as well as to participation in general. This education should take place as soon as possible. Research suggests that interventions to change cognitions should focus on patients in an early stage of the illness process [44]. The best moment to offer interventions to alter maladaptive beliefs in patients with CKD seems to be in the pre-dialysis phase (preferably even before CKD stage IV) or at the start of renal replacement therapy, in which patients are most susceptible to change. In the Netherlands, psychological support is not yet a primary area of attention in pre-dialysis care and renal care in general. It is therefore important that interventions which focus on these psychological concepts are developed [45].

## Competing interests

The PREPARE-2 study is an independent academic study designed and carried out by the Department of Clinical Epidemiology from the Leiden University Medical Center in collaboration with the Hans Mak Institute (Naarden, The Netherlands) and the participating centers. This study was funded by unrestricted grants from Amgen BV (Breda, The Netherlands), Abott (Hoofddorp, The Netherlands), Genzyme (Naarden, The Netherlands), Shire (Hampshire, United Kingdom), The Dutch Kidney Foundation (Bussum, The Netherlands) and the Leiden University Medical Center (Leiden, The Netherlands).

The authors have had no involvements that might raise the question of bias in the work reported or in the conclusions, implications, or opinions stated. The results presented in this paper have not been published previously in whole or part, except in abstract format.

## Authors' contributions

DJ, DG, MR: have made substantial contributions to acquisition of data, or analysis and interpretation of data; DJ, DG, MR, MH, AK, EB and FD: have made substantial contributions to conception and design; DJ: has drafted the manuscript and DG, MR, MH, AK, EB and FD: have been involved in revising the manuscript critically for important intellectual content. All authors have given final approval of the version to be published.

## Pre-publication history

The pre-publication history for this paper can be accessed here:

http://www.biomedcentral.com/1471-2369/11/35/prepub
